# COVID-19 stressors for Hispanic/Latino patients living with type 2 diabetes: a qualitative study

**DOI:** 10.3389/fcdhc.2023.1070547

**Published:** 2023-04-28

**Authors:** Myia S. Williams, Edgardo Cigaran, Sabrina Martinez, Jose Marino, Paulina Barbero, Alyson K. Myers, Ralph J. DiClemente, Nicole Goris, Valeria Correa Gomez, Dilcia Granville, Josephine Guzman, Yael T. Harris, Myriam Kline, Martin L. Lesser, Amgad N. Makaryus, Lawrence M. Murray, Samy I. McFarlane, Vidhi H. Patel, Jennifer Polo, Roman Zeltser, Renee Pekmezaris

**Affiliations:** ^1^ Department of Medicine, Division of Health Services Research, Northwell Health, Manhasset, NY, United States; ^2^ Donald and Barbara Zucker School of Medicine at Hofstra/Northwell, Hempstead, NY, United States; ^3^ Feinstein Institute for Medical Research, Northwell Health, Manhasset, NY, United States; ^4^ Northwell Health, Manhasset, NY, United States; ^5^ Department of Medicine, Division of Endocrinology, Albert Einstein/Montefiore, Bronx, NY, United States; ^6^ Department of Social and Behavioral Sciences, New York University (NYU) School of Global Public Health, New York, NY, United States; ^7^ Hispanic Counseling Center, Hempstead, NY, United States; ^8^ Department of Medicine, Division of Endocrinology, North Shore University Hospital, Manhasset, NY, United States; ^9^ Department of Cardiology, Nassau University Medical Center, East Meadow, NY, United States; ^10^ Annie E. Casey Foundation Children and Family Fellowship, Baltimore, MD, United States; ^11^ Division of Infectious Disease, College of Medicine, SUNY-Downstate Health Sciences University, Brooklyn, NY, United States

**Keywords:** health disparities, COVID-19, telemonitoring, racial/ethnic minorities, health equity, stakeholder engagement

## Abstract

**Background and aim:**

During the early stages of the COVID-19 pandemic, nationwide lockdowns caused disruption in the diets, physical activities, and lifestyles of patients with type 2 diabetes. Previous reports on the possible association between race/ethnicity, COVID-19, and mortality have shown that Hispanic/Latino patients with type 2 diabetes who are socioeconomically disadvantaged are disproportionately affected by this novel virus. The aim of this study was to explore stressors associated with changes in diabetes self-management behaviors. Our goal was to highlight the health disparities in these vulnerable racial/ethnic minority communities and underscore the need for effective interventions.

**Methods and participants:**

Participants were enrolled in part of a larger randomized controlled trial to compare diabetes telehealth management (DTM) with comprehensive outpatient management (COM) in terms of critical patient-centered outcomes among Hispanic/Latino patients with type 2 diabetes. We conducted a thematic analysis using patient notes collected from two research nurses between March 2020 and March 2021. Two authors read through the transcripts independently to identify overarching themes. Once the themes had been identified, both authors convened to compare themes and ensure that similar themes were identified within the transcripts. Any discrepancies were discussed by the larger study team until a consensus was reached.

**Results:**

Six themes emerged, each of which can be categorized as either a source or an outcome of stress. Sources of stress associated with the COVID-19 pandemic were (1) fear of contracting COVID-19, (2) disruptions from lockdowns, and (3) financial stressors (e.g., loss of income). Outcomes of COVID-19 stressors were (1) reduced diabetes management (e.g., reduced diabetes monitoring and physical activity), (2) suboptimal mental health outcomes (e.g., anxiety and depression), and (3) outcomes of financial stressors.

**Conclusion:**

The findings indicated that underserved Hispanic/Latino patients with type 2 diabetes encountered a number of stressors that led to the deterioration of diabetes self-management behaviors during the pandemic.

## Background

Previous reports on associations between race/ethnicity, COVID-19, and mortality have shown that, compared with white individuals, Hispanic/Latino (H/L) individuals, particularly those who are socioeconomically disadvantaged, are disproportionately affected by this novel virus (1–4). For example, at the start of the pandemic, in New York City, once the epicenter of COVID-19, H/Ls account for 28% of fatalities despite representing 22% of the population (5, 6). Similarly, one study showed that between 2019 and 2020 the life expectancy of H/Ls declined by 3 years, the largest decrease of any racial/ethnic group in the United States (4, 7). Meanwhile, a more recent report by the Centers for Disease Control and Prevention (CDC) found that in August 2022, relative to their percentage in the population (18.9%), H/Ls made up 24.8% of COVID-19 cases and 15.8% of COVID-19 deaths in the United States (8).

While COVID-19 led to a major decline in life expectancy and an increase in death rates among the H/L population, diabetes was reported to be the third leading cause of death contributing to health disparities within the H/L community (4). Compared with white Americans, H/Ls are 66% more likely to develop type 2 diabetes (T2D) (9–11) and are 2.4 times more likely to die from COVID-19 (4, 12). In addition, H/Ls are known to have worse outcomes and lower levels of diabetes management than their white counterparts (4, 11). Several reports have shown that H/L patients with diabetes have a 50%–100% greater illness burden (4, 9, 11), a 41% higher rate of end-stage renal disease (11, 13), and an 84% higher rate of diabetic retinopathy than their white counterparts, and are at twice the risk of lower extremity amputation (11, 14, 15). Furthermore, research shows that, compared with white individuals, H/L adults receive lower-quality diabetes-related healthcare (e.g., less frequent measurement of hemoglobin A1C (HbA1c)) (16), which is even more pronounced in H/L patients with limited English proficiency and those who are not US citizens (16, 17). Other factors, such as a lack of patient–provider concordance, cultural values and beliefs (e.g. fatalism), and attitudes toward diabetes and treatments, are documented influences on the adherence to and outcomes of diabetes management in H/L individuals (17–20).

In addition to T2D being most prevalent in H/L communities, the disparities experienced by H/Ls from COVID-19 and diabetes are exacerbated by the already existing sociocultural, psychosocial, and environmental vulnerabilities (1, 4, 12). H/Ls face increased challenges in access to care in terms of COVID-19 and T2D testing and treatment services, have higher rates of uninsured and underinsured access to T2D treatment and care than their white counterparts, have a fear of using healthcare and social services because of immigration status, and face language barriers that result in a reduction in access to and understanding of educational materials for treatment (1, 4, 21). Additionally, H/Ls have higher rates of job loss or are employed in positions (e.g., essential/frontline services) that do not offer paid leave, opportunities to work from home, or insurance (4). They are also more likely to live in overcrowded housing and/or multigenerational family homes and in densely populated areas, all of which factors limited H/L individuals’ ability to comply with mitigation strategies and increased the risk of exposure to and infection of COVID-19 (4, 12).

The factors above also complicated diabetes management for H/L individuals who were faced with financial and economic hardship, which created barriers to accessing quality healthcare and affording medication, diabetes supplies, and nutritious food to maintain a healthy diet during the COVID-19 pandemic (1, 4, 21). In addition, confinement measures to prevent the spread of the virus led to a reduction in physical activity and modified daily routines, which altered glycemic control and thus had an adverse impact on the mental health and coping capabilities of vulnerable H/L individuals (1, 4, 12). Overall, people with T2D reported eating more, exercising less, and having higher levels of stress and depression than in the pre-pandemic period (22, 23). For H/L populations with strong family ties (19, 20), lockdowns and shelter-in-place orders meant less access to social support networks, which could have exacerbated mental health effects (22). Depression and diabetes distress are two conditions that are known to affect people with T2D, which can also directly increase the risk of mortality, work absenteeism, and poor diabetes management and health outcomes (24, 25). H/Ls with T2D are known to experience, on average, higher rates of both depression and diabetes distress (26).

Home telemonitoring (HTM) is considered an effective tool in improving clinical, quality-of-life, and healthcare cost outcomes for patients living with T2D (9, 11). Many HTM platforms use Bluetooth technology to provide up-to-the-minute updates on patients’ vital signs (e.g., blood sugar, weight, blood pressure, and heart rate), which clinicians can closely monitor. HTM also allows clinicians to initiate video visits with their patients to discuss treatment, provide educational materials, and answer any questions related to diabetes management (11). Previous research, including meta-analyses of randomized controlled trials, has shown significant improvements in T2D management (27) and reductions in glucose levels (28–30).

Despite potential clinical benefits (31), HTM utilization, access, and participation are particularly low among underserved H/L patients with T2D, largely because of practical barriers (e.g., working schedules) and health system barriers (9, 11). Given the unique sociocultural experiences (e.g., multigenerational households), multiple jobs (e.g., frontline/essential services), and barriers (e.g., access to care) encountered by H/L patients with T2D that were exacerbated by the COVID-19 pandemic, it is imperative that interventions and diabetes management programs are specifically tailored to meet the cultural and socioeconomic needs of this vulnerable community (9, 11). However, few studies have examined how HTM can be tailored to the specific needs of H/Ls with T2D arising from their cultural and socioeconomic circumstances and low health literacy (11). During the COVID-19 pandemic, HTM was shown to be effective in the general population; however, it is unclear how HTM can be culturally tailored to meet the needs of vulnerable H/L people with T2D. Therefore, our study sought to fill the critical knowledge gaps identified by meta-analyses and observational studies by exploring stressors associated with changes in diabetes self-management behaviors (23) and examining the impact of HTM on diabetes management in vulnerable H/L people with T2D during the pandemic. Our goal was to highlight the health disparities in these vulnerable racial/ethnic minority communities and underscore the need for effective interventions.

## Methods

### Ethics

Ethics approval was granted by the Feinstein Institute of Medical Research Institutional Review Board (#19-0002 and #21-0292). Data were collected with the written informed consent of participants involved in a larger randomized clinical trial (RCT) (NCT03960424).

### Study design

The study used a phenomenological qualitative design to collect data from the notes taken by research study nurses during interactions with patients from March 2020 to March 2021. These in-depth, semi-structured notes provided rich and detailed insights regarding the COVID-19 stressors experienced by H/L patients with T2D and how those stressors impacted their daily routine and subsequent diabetes management during the COVID-19 pandemic. This study was a substudy conducted in the context of a larger multiphase mixed-methods study, “diabetes telehealth management in the H/L population with type 2 diabetes” (11), which aimed to compare diabetes telemonitoring (DTM) with comprehensive outpatient management (COM) or usual care in terms of critical patient-centered outcomes among H/L patients with T2D. The methodology for the study (11) was grounded in the self-efficacy (SE) theoretical framework. SE beliefs are patient thought patterns that influence health behavior, including whether behaviors are initiated and effort is used and sustained while experiencing impediments to progress. The American Diabetes Association (ADA) recognizes SE as critical in improving T2D management and recommends SE assessment in interventions (11, 32). Increased SE predicts improved glucose management (GM) and is particularly important in disadvantaged patients, who must overcome many barriers to T2D management (11, 32).

### Participants

Participants were enrolled in part of a larger randomized controlled trial to compare DTM (14 women, seven men; average age 60 years) with COM (nine women, five men, average age 60 years) in terms of critical patient-centered outcomes among H/L patients with T2D. Participants were identified through convenience sampling, a non-probability sampling method in which members of the population who meet a particular criterion, in this case, accessibility, are included for the purpose of the study (33, 34). Participants were self-identified H/L patients with a diagnosis of T2D, alone or in combination with other chronic conditions (e.g., heart disease), were aged ≥ 18 years, and were English or Spanish speakers.

### Data collection

Qualitative data were collected during weekly televisits (DTM arm) or monthly phone calls (COM arm) with one of two research nurses, both of whom were women and identified as H/L. During the video visits (DTM only), patients and research nurses would discuss diabetes management and any concerns that the patients had. COM patients were asked to simply report their healthcare utilization. To ensure consistency and uniformity across both nurses, the research nurses’ notes and questions were based on standard questions asked during routine patient visits. Both groups routinely had their hemoglobin A1c (HbA1c) levels checked. For patients in the DTM arm, the research nurses were able to monitor vital signs that were uploaded by the patient daily. The research nurses recorded all notes and information from the conversations with the patients. The nurses’ progress notes included questions that were guided by a protocol whereby they were to document responses from participants. Those questions were rooted in the SE framework to engage, encourage, and empower participants to reflect upon and discuss their daily metrics from the previous week. The purpose of this was to collaboratively identify strategies such as goal setting and planning to facilitate self-management of T2D. In addition, key variables in managing T2D, such as diet, physical activity, and self-monitoring of blood glucose, were discussed during each telehealth visit in the participant’s preferred language, using simple words commonly used and understood by each participant. The research nurses used Institutional Review Board (IRB)-approved and Health Insurance Portability and Accountability Act (HIPAA)-compliant measures to maintain confidentiality, privacy, and data security. Although some data were collected in Spanish by the bilingual nurses, all notes were recorded in English and entered and stored in a restricted password-protected drive and in RedCap.

### Data analysis

We used an inductive thematic analysis (35) to analyze the research nurses’ notes. Based on Braun and Clarke’s six phases of thematic analysis, to optimize the credibility, transferability, and dependability of results, the investigators utilized researcher triangulation and peer debriefing and documented the trail of decisions made during the analysis and rationale (35). In phase 1, two members of the research team MSW and EC independently read the notes multiple times to become familiar with the data and to capture the overall perspectives of the participants. In phase 2, the two researchers focused on patterns noted and observed in the data to generate an initial set of codes. In phase 3, the researchers sorted and collated the codes into potential themes. Next, in phase 4, the researchers reviewed the themes to identify coherent patterns and assess whether those patterns were aligned with the specific aims of this substudy. In phase 5, the researchers met to discuss their individual themes and subthemes, then “defined and refined” when necessary. To resolve disagreements, the researchers engaged in open discussions and revisions until a consensus was reached. When a consensus was not reached, disagreements were moderated by the principal investigator (RP) of the larger study. Finally, in phase 6, all members of the research team met to discuss and finalize the themes. The final themes reflected the experiences of the participants based on the research notes as well as the purpose of the research aims.

## Results

The COVID-19 stressors faced by H/L patients with T2D were categorized into six themes, each of which was categorized as either a source or outcome of stress. Illustrative quotations from patients are presented to highlight key study findings. It should be noted that, while we have two distinct arms in the study, both arms experienced the same COVID-19 stressors.

### Sources of stress associated with the COVID-19 pandemic

We identified stressors as related to the COVID-19 pandemic if they were demanding events and stimuli that were specifically related to/associated with the COVID-19 pandemic, that is, they were events and stimuli that did not exist prior to the pandemic but came about as a result of the pandemic (36).

#### Theme 1: fear of contracting COVID-19

Due to diabetic complications, several patients reported a fear of contracting COVID-19 and as a result remained isolated in their homes, avoiding crowds and adhering to CDC guidelines to reduce the risk of contracting COVID-19:

“Subject denies physical activity over the past, which subject attributed to stressors related to COVID-19, subject states he prefers to stay home and avoid crowded places at this time. Subject reports stressors related to COVID-19. Subject states as per CDC recommendations he has been taking precautions to reduce his risk of getting sick. Subject states he is currently avoiding crowds and staying home as much as possible. [sic]”

Fear of contracting COVID-19 also resulted in patients foregoing medical attention, even when they were sick, and it was recommended by their doctor that they present to the ER:

“Subject reports she called and spoke with her primary care provider today regarding symptoms and fall stated above, and was advised to go to the ED [emergency department] for an evaluation. Subject denies going to the ED as instructed by provider, which she attributed to fear of contracting COVID-19. [sic]”

Unfortunately, patients lost their lives because of fear of contracting COVID-19 while at the hospital:

“Husband states subject was swabbed for COVID-19 after he tested positive, and her result came back negative. Husband states subject started to experience headache, fever, chills, nausea, vomiting, fatigue, loss of appetite, and shortness of breath (SOB) after testing negative for COVID-19. Husband states subject had a telehealth visit with primary care physician (PCP) and was advised to go to ED with worsening symptoms, but subject declined. Husband states subject’s repeat COVID-19 swab result came back positive. Husband states subject verbalized to her sister that she was afraid of going to the hospital and that she was giving up on life, therefore, declined to go to the ED or Monoclonal antibody (MAB) treatment. Subsequent telephone call placed at scheduled time and spoke with Husband. He states subject passed away at home this AM. Husband states subject was SOB in AM but declined to go to ED. Husband states he then found subject unresponsive and called EMS. [sic]”

#### Theme 2: disruptions from lockdowns

At the initial stages of the pandemic, a number of patients either left the country or were out of the country/state and could not make it back because of lockdowns and stay-at-home orders. As a result, COVID-19 brought on the additional stressor of a lack of supplies and access to medication:

“Subject states due to the unexpected length of stay he is running out of blood glucose monitoring supplies and is requesting additional supplies to be sent to his residence in Florida. Study recruiter informed subject that additional supplies will be sent. [sic]”

COVID-19 also led to the cancellation of a number of non-essential procedures and doctors’ visits, which also caused additional stress, particularly if patients needed to see their doctor but could not because of COVID-19 restrictions enforced by healthcare systems:

“Subject report pain and numbness in his both legs. Subject stated that doctor aware but unfortunately cannot see him until COVID-19 is resolved. [sic]”

#### Theme 3: financial stressors (e.g., loss of income)

The COVID-19 pandemic had a significant impact on the financial and economic livelihood of our patient population. Some patients reported that they lost their job and faced significant economic hardships:

“Subject reports stressors related to COVID-19. Subject states both spouse and subject were laid off from work unpaid approximately one week ago. Subject reports financial hardship over the past week. [sic]”

Patients also reported that the financial hardships they encountered as a result of COVID-19 were also affecting their physical health:

“Subject complains of chest pain, SOB, anxiety, stressors, sadness, and disappointment. Subject states chest pain and SOB were only present one day approximately two weeks ago, which subject attributed to financial hardship, food insecurities, unemployment, and living situation (lives in an apartment building and not able to get out as much as she would like to secondary to COVID-19). Symptoms resolved by walking and speaking with a friend. [sic]”

### Outcomes of COVID-19 stressors

The stressors related to COVID-19 resulted in reduced diabetes management and suboptimal outcomes (e.g., reduced mental health outcomes) in our patients.

#### Theme 1 reduced diabetes management (e.g., reduced diabetes monitoring and physical activity)

One of the biggest stressors faced by patients was a reduction in diabetes management such as reduced physical activity and glucose monitoring, which stemmed from stressors associated with COVID-19, contracting COVID-19, and lockdowns and shelter-in-place orders that prevented individuals from being active. While both groups noted a reduction in diabetes management, it was noted more often by patients in our DTM arm reported than in our COM arm. One research nurse noted the following in relation to a patient:

“Subject reports she forgot to measure blood glucose levels and weight over the past week due to stressors related to two deaths in the family over the past week related to complications of COVID-19. [sic]”

Another patient attributed his lack of diabetes management to stressors associated with having contracted COVID-19 twice:

“Subject states at this moment that he is not measuring his blood glucose level, blood pressure and weight as recommended by his provider because he is very stressed out about being diagnosed for a second time with COVID-19. [sic]”

Other patients reported that having family members at home during shelter-in-place orders was also impacting the number of times they measured their glucose levels:

“Subject states she has been measuring blood glucose levels 1–2 times a day (fasting, breakfast postprandial, and dinner postprandial, time of measure varies daily) in the past 2 weeks due to stressors related to COVID-19. Subject states amid the COVID-19 pandemic her children and family are back home and she is too busy or forgets to measure blood glucose levels 3 times a day. [sic]”

Owing to the lack of physical activity and quality food, several patients also experienced hypoglycemic and hyperglycemic episodes:

“Trend of blood glucose readings were reviewed and are within baseline over the past week, with hyperglycemia, asymptomatic, which subject attributed to food choices (tortilla, does not remember other foods) and low levels of physical activity. Subject attributed changes to diet and level of physical activity to stressors related to COVID-19 and adhering to COVID-19 guidelines. [sic]”

Lastly, several participants also indicated that, due to the stay-at-home orders, they were unable to leave their homes and take part in any form of physical activity:

“Subject denies physical activity over the past week due to stressors related to the COVID-19 and adherence to social distancing guidelines as recommended, therefore avoiding leaving home. [sic]”

#### Theme 2: suboptimal mental health outcomes (e.g., anxiety and depression)

Several patients reported that they were struggling with their mental health and experienced anxiety and depression as a result of the pandemic. This theme was noticeably more common in our COM arm than in our DTM arm:

“Subject states she went to NSUH ED with palpitations, high blood pressure, dizziness, and nausea. Subject states she got blood work and EKG [electrocardiogram] done and was discharged home and advised to follow up with PCP [primary care provider]. Subject denies getting admitted to the hospital. Subject states she followed up with PCP and got sertraline for anxiety. Subject states she has been taking sertraline as prescribed by provider with positive effect. [sic]”

Patients also reported feeling anxious or depressed because of unemployment or other factors such as fear of contracting COVID-19 or challenges that they had faced as a result of COVID-19:

“Subject states she followed up with PCP with symptoms of depression and got script for antidepressants and will follow up with social worker. Subject states she did not start taking the medication yet. Subject attributed symptoms of depression to financial hardship secondary to COVID-19. [sic]”

While most of our patients who reported having mental health stressors reported finding ways to cope, they declined opportunities to seek professional help:

“Subject states she has been coping with the grief by walking and talking to her sister with positive effect. In the past subject was advised by provider to follow-up with a counselor but subject declined. Provided subject with the contact information to the [XXX] Crisis Center. [sic]”

Several of our patients reported negative mental health outcomes as a result of fear of contracting COVID-19, actually contracting COVID-19, or an inability to control their interactions with others who may have been in contact with individuals who had COVID-19:

“Subject stated that in this moment, he feels a lot of stress because at his wife’s job five people were COVID-19 positive and his wife has some symptoms and for that reason both had been tested for COVID-19 but they are waiting for their results. [sic]”

Lastly, and of note, several of our patients reported that poorer mental health outcomes (e.g., being stressed from COVID-19) resulted in reduced diabetes management because they did not want to deal with anything else:

“Subject states at this moment he is not measuring his blood glucose level, blood pressure, and weight as recommended by provider, because he is very stressed out about COVID-19 and does not want to deal with anything else. [sic]”

#### Theme 3: outcomes of financial stressors (e.g., food and housing insecurity)

During the COVID-19 pandemic, several of our patients and other financial contributors in their households lost their sources of income, which led to a significant ripple effect in terms of unmet needs such as food and housing security, which in turn led to mental health issues:

“Subject complains of anxiety and stressors related to financial hardship and issues at home. Subject states the house where she currently resides was sold to another landlord and she has to move, but she has not been able to find an apartment. Subject states she lost power and access to hot water at home for two days. Subject states the hot water and power came back on today. Subject states she had to get rid of all her food as the food got spoiled in the refrigerator due to losing power for two days. Subject states she followed up with provider post hospital discharge and discussed anxiety and stressors with provider and got sertraline and referral for counseling. [sic]”

In a similar situation, another patient reported facing difficulty with paying rent and the possibility of being evicted from her home:

“Subject states her spouse stopped working since he was diagnosed with testicular cancer and subject lost her job due to COVID-19. Subject states she is struggling to find work and has not been able to pay her bills or rent. Subject states landlord is upset and is requesting subject and family vacate the apartment due to inability to pay rent. Subject states she explained the situation to the landlord but he continues to ask for the rent money. Subject denies suicidal or homicidal ideation at this time. [sic]”

Other participants reported seeking outside assistance to gain food and to know their rights:

“Subject states she went to social services and got food stamps and was advised she cannot get evicted from her apartment for not paying the rent at this time. Subject states she feels better today. Subject advised to contact provider or go to ED/urgent care (UC) with worsening symptoms. Subject agreed to continue to follow up with provider and social worker regarding depression. [sic]”

In a similar situation, another participant reported getting help from their church:

“Subject states, unfortunately I have to end video call because I have to go to pick up free food and grocery baskets that the Church is giving to us due to COVID-19. [sic]”

## Discussion

The literature suggests that systemic inequities in social and economic conditions can prevent people from accessing resources that are vital to preparing for and responding to public health emergencies, such as a pandemic or natural disaster (37–39). In the current study, H/Ls with T2D reported reduced diabetes management as a result of complying with COVID-19 protocols (e.g., lockdowns) and a lack of access to resources that stemmed from financial hardship and which lead to unmet needs (e.g., housing and food insecurity). Patients also reported a significant increase in levels of anxiety and depression, which stemmed from the pandemic, as well as in COVID-19-related stressors such as fear of contracting the virus. The results from this study can help to create culturally tailored home-telemonitoring interventions for vulnerable and underserved H/L communities by anticipating these situations and making accurate information and resources readily available to this population.

Our study found a significant reduction in diabetes management among our patient group during the pandemic. This decrease was partially a result of the lockdowns and shelter-in-place protocols implemented to stop the spread of the virus. Notably, our patients were aware of the complications of having COVID-19 and diabetes, and as a result, were taking the necessary precautions to ensure that they did not become infected. However, these very same mitigation strategies often resulted in a reduction in diabetes management. In addition to having to shelter in place, patients reported experiencing other stressors associated with COVID-19 (e.g., living in a multigenerational household) that prevented them from measuring their glucose as recommended by their healthcare provider. Unfortunately, some of our participants were disproportionately affected by food insecurity during the COVID-19 pandemic, which they attributed to unexpected lay-offs, stressors, and reduced income levels. Consequently, participants reported drastic changes in their diet/quality of food, lack of exercise, and self-adjusting their diabetes medications, which may have contributed to hyperglycemia and hypoglycemia. For example, some participants reported administering more or less insulin than prescribed by their provider. A recent study found that the COVID-19 pandemic significantly impacted the health habits (food and physical activity) of patients with T2D (22).

Notably, we found that more DTM patients than COM patients reported a lack of physical activity and unhealthy eating habits. This may be because of the frequent contact that our research nurses had with our DTM patients and their ability to monitor patients’ vital signs on a regular basis and therefore notice patterns in their diabetes management that could be rectified in a timely manner; in contrast, we could contact patients in the COM arm only once a month *via* phone and we did not have access to their vital signs. This point underscores the clear need for easy-access systems, such as telehealth, that can enable continuous monitoring of patients during a pandemic while minimizing the exposure of both clinical staff and patients (40). Additionally, telehealth can level the playing field for H/L patients from poor socioeconomic backgrounds, who may not have the financial resources to travel to in-person visits (40). Previous research shows that during the COVID-19 pandemic, platforms such as Skype and FaceTime increased access to telehealth as a modality of treatment for historically minoritized patients who were otherwise unable to afford more expensive applications of telehealth or a smartphone or computer (23, 40).

Another theme that emerged was COVID-19 stressors, which are stressors specifically related to the COVID-19 pandemic (e.g., fear of contracting COVID-19). One of the most noticeable subthemes was that, for fear of contracting COVID-19, patients declined to access medical services or to visit doctors. Unfortunately, we lost patients because of this fear. In addition, medical facilities were restricted and there were limited possibilities to access facilities for non-essential procedures (23). We also found that the participants in our study reported negative psychological outcomes (e.g., anxiety and depression) from both COVID-19 stressors and ripple effects (e.g., loss of job and housing insecurity). While the profound mental impact of COVID-19 is likely to affect all individuals regardless of race and class, evidence from past disasters and public health emergencies suggests that individuals from racial/ethnic minority groups and socioeconomically disadvantaged groups are likely to experience more negative psychological outcomes (41, 42). The recommended mitigation and prevention strategies may have had unintended consequences such as affecting access to vital resources and social support networks, which have been proven to be instrumental in coping with stress, particularly for members of the H/L community (43, 44). This could lead to the adoption of poor coping mechanisms, such as increased overeating and reduced physical activity. Consequently, if COVID-19 stressors and financial stressors remain significantly unaddressed among vulnerable racial/ethnic minority communities, mental health issues will significantly increase and exacerbate disparities (42, 44).

Addressing sociocultural status is critical for H/L patients who are faced with challenges in accessing healthcare that meets their needs. Emerging research in racial/ethnic health disparities and addressing unmet social needs indicates that innovative and effective interventions are needed in underserved H/L communities, given that (1) H/Ls are disproportionately affected by COVID-19 and diabetes, (2) traditional healthcare system approaches rarely recognize and screen for cultural factors that influence H/L and other racial/ethnic minorities’ health behavior (45–48), and (3) there is a dearth of research examining effective strategies for culturally tailored interventions for H/L patients. Previous research, including ours (40, 41, 49), highlights the need for easily accessible, culturally competent, race-conscious interventions that actively address the psychosocial factors that influence an individual’s mental and physical wellbeing (49). One strategy that researchers and practitioners alike agree on is that culturally and ethnically congruent medical professionals should be at the forefront in the dissemination of accurate and timely information to historically marginalized communities, particularly since there is a history of mistrust and thwarted relationships between minority patients and healthcare systems (41). Our combined patient–healthcare provider and web-based intervention focusing on addressing both unmet health-related needs and well-being in this vulnerable community addresses this.

Our study is not without limitations. First, our small sample size makes it difficult to generalize across other populations. In addition, our research nurses had more contact with our DTM arm than with our COM arm, which may have skewed data toward the experiences of the DTM than the COM arm. Our findings are therefore representative of the small sample size, particularly for the DTM arm, and may not be inclusive of all of the lived experiences of other members of the H/L community. Second, our patient notes were rather succinct, and our study could have been further enriched if we had conducted interviews directly to learn more about the experiences and challenges during the COVID-19 pandemic of H/L patients living with T2D. Third, our study was unable to determine causality because it was a qualitative study. We did not assess whether patients had anxiety or depression (conditions that are historically associated with diabetes) prior to the COVID-19 pandemic. Further research should therefore focus on designing quasi-experimental or experimental designs that can determine causality.

## Data availability statement

The original contributions presented in the study are included in the article/supplementary material. Further inquiries can be directed to the corresponding author.

## Ethics statement

The studies involving human participants were reviewed and approved by the Feinstein Institute of Medical Research. The patients/participants provided their written informed consent to participate in this study.

## Author contributions

MW, conceptualization and original manuscript draft; MW and EG coded data and conducted analyses; ED, JM, VC, and NG recruited participants; SM and PB are the research nurses on the study and recorded patient data; MW and RP, methodology. All authors contributed to reviewing and editing the manuscript. All authors contributed to the article and approved the submitted version.
